# Investigating Transfer Learning in Noisy Environments: A Study of Predecessor and Successor Features in Spatial Learning Using a T-Maze

**DOI:** 10.3390/s24196419

**Published:** 2024-10-03

**Authors:** Incheol Seo, Hyunsu Lee

**Affiliations:** 1 Department of Immunology, Kyungpook National University School of Medicine, Daegu 41944, Republic of Korea; 2 Department of Physiology, Pusan National University School of Medicine, Yangsan 50612, Republic of Korea; 3 Research Institute for Convergence of Biomedical Science and Technology, Pusan National University Yangsan Hospital, Yangsan 50612, Republic of Korea

**Keywords:** reinforcement learning, T-maze transfer learning, hyperparameter tuning, robustness, predecessor features, noisy observation

## Abstract

In this study, we investigate the adaptability of artificial agents within a noisy T-maze that use Markov decision processes (MDPs) and successor feature (SF) and predecessor feature (PF) learning algorithms. Our focus is on quantifying how varying the hyperparameters, specifically the reward learning rate ($\alpha_{r}$) and the eligibility trace decay rate (λ), can enhance their adaptability. Adaptation is evaluated by analyzing the hyperparameters of cumulative reward, step length, adaptation rate, and adaptation step length and the relationships between them using Spearman’s correlation tests and linear regression. Our findings reveal that an $\alpha_{r}$ of 0.9 consistently yields superior adaptation across all metrics at a noise level of 0.05. However, the optimal setting for λ varies by metric and context. In discussing these results, we emphasize the critical role of hyperparameter optimization in refining the performance and transfer learning efficacy of learning algorithms. This research advances our understanding of the functionality of PF and SF algorithms, particularly in navigating the inherent uncertainty of transfer learning tasks. By offering insights into the optimal hyperparameter configurations, this study contributes to the development of more adaptive and robust learning algorithms, paving the way for future explorations in artificial intelligence and neuroscience.

## 1. Introduction

Within the context of the considerable attention paid to transfer learning in the artificial intelligence (AI) field, reinforcement learning (RL) has come to the forefront given its potential to adapt previously acquired knowledge to new related tasks or environments [1]. Significantly, RL necessitates that agents acquire optimal decision-making strategies through iterative interactions with the environment, raising the issue of sample efficiency [2]. By leveraging the capabilities of transfer learning, RL agents can substantially diminish the temporal and computational resources needed for them to adapt to new circumstances, thereby enhancing the efficacy and resilience of their learning algorithms. Broadly speaking, the implementation of transfer learning in RL enables RL agents to decompose a singular task into several constituent subtasks and subsequently leverage the knowledge amassed from these subtasks to expedite the learning process for related tasks [3,4,5]. Nonetheless, in instances where the subtasks are not correlated, transfer learning remains unattainable. Therefore, in this paper, we focus on distinct scenarios with different reward functions but uniform subtask dynamics.

As a crucial brain structure involved in memory and knowledge transfer, the hippocampus has been extensively studied in order to understand its role in facilitating learning across various cognitive domains [6,7,8,9,10]. This process of knowledge transfer shares similarities with the concept of transfer learning in RL algorithms, and successor representation (SR) has emerged as an explainable algorithmic theory that may provide insights into the function of the hippocampus in transfer learning [7,11,12,13]. In alignment with the neural activity observed in the hippocampus, SR is a predictive model of the environment that emphasizes the relationships between current states and potential future states, with noteworthy implications for transfer learning in RL algorithms. By incorporating SR as an underlying mechanism, RL agents can potentially leverage the same principles that govern hippocampal function in knowledge transfer, resulting in more efficient and robust learning algorithms. This connection between neural correlates and RL provides researchers with the opportunity to explore the application of SR to RL further and investigate its potential to enhance the capability of transfer learning.

Through linear approximation, successor features (SFs) extend SR algorithms and enhance their applicability in transfer learning by addressing the reward dynamics and enabling them to learn from environmental structures independently [14,15,16,17,18]. With the employment of a temporal difference (TD) learning algorithm, integrating an eligibility trace into SFs becomes a seamless extension [2]. Indeed, investigations have revealed that SFs enhanced with an eligibility trace, that is, predecessor features (PFs), surpass the performance of standalone SFs [19,20]. PFs are specifically designed to assess the influence of an agent’s past state on its current state, underscoring the relationship between historical experiences and the present circumstances. With regard to temporal difference (TD) learning rule algorithms, SFs adhere to a constrained learning rule variant, TD (λ=0), while a more comprehensive form of the learning rule, TD(λ), applies to PFs. In spite of the extensive research on SFs within the realm of transfer learning, to the best of our knowledge, the application of PFs has been examined notably less.

In RL, the hyperparameters play a pivotal role in transfer learning tasks, determining the success or failure of knowledge transfer between tasks. For success, the appropriate hyperparameter configurations must be identified, as they directly influence a learning agent’s ability to adapt to and generalize across diverse settings. Inappropriate hyperparameter settings may hinder an agent’s ability to recognize and exploit commonalities between tasks or environments, leading to suboptimal performance and reduced learning efficiency [3]. Conversely, well-tuned hyperparameters enable agents to effectively capitalize on shared structures and rapidly adapt to novel situations, thereby reducing the time and computational resources needed for high performance. However, most of the existing research has focused on developing novel transfer learning techniques and evaluating their performance in specific tasks and environments [3,4,5]. Although hyperparameter optimization is evidently important in transfer learning, the current state of the research in this area remains limited.

Previous works have investigated the sensitivity of deep neural networks to noise and quality distortions such as blur and pixelation to observe their effect on deep learning models, particularly in image recognition tasks [21,22,23]. These studies have shown that noise, such as that encountered by RL agents navigating complex environments, can significantly degrade the model performance. Equally, given the well-documented problem of working with small amounts of data in intelligent systems, recent research has emphasized the need for robust models that can operate under data sparsity and variability [24]. Our approach addresses these issues by optimizing adaptive learning in noisy environments and limited data scenarios. Advanced approaches such as transformer-based few-shot learning have shown promise in dealing with noisy labels and varying conditions, demonstrating the potential of adaptive algorithms in complex environments [25]. Furthermore, by combining a conditional variational autoencoder and generative adversarial networks (GANs), Li et al. [26] developed a zero-shot detection method for unmanned aerial vehicle sensors. This approach represented an innovative strategy for identifying faults with minimal training data, showcasing the potential of advanced machine learning models to handle data complexity and sparsity. Overall, these studies highlight the importance of addressing noise in sensor data to improve the resilience and adaptability of RL agents in noisy environments.

In this investigation, we focus on the critical role of hyperparameter tuning in enhancing the effectiveness of transfer learning strategies, particularly concerning PF and SF algorithms. The real-world implications of RL underscore the need to assess the performance of these algorithms in noisy environments and their potential to augment transfer learning. We previously identified a complex relationship between noise levels and PF performance that was notably influenced by the λ parameter [27]. Motivated by these findings, here, we seek to explore how the hyperparameters can impact the efficiency of transfer learning further, especially in noisy conditions, and to pave the way for more adaptive and robust RL applications. This study offers novel contributions by (1) evaluating the impact of the hyperparameters on adaptive behavior in noisy spatial learning environments using a T-maze; (2) introducing an SF- and PF-based framework for the comparison and quantification of sensitivity to noise and adaptation; (3) and identifying the most robust hyperparameter configurations under noisy and variable conditions.

In this study, we evaluated the adaptive performance in a noisy T-maze using a Markov decision process (MDP) framework and a PF learning algorithm. Section 2 discusses the noise-related challenges in RL, the benefits of enhancing its efficiency using transfer learning, and the crucial role of hyperparameter optimization in improving the performance and robustness of RL algorithms. Section 3 outlines our methodology, including the T-maze setup, hyperparameters, and performance metrics. Section 4 details the results of testing 25 combinations of hyperparameters and their reward acquisition, step lengths, and adaptability. Section 5 discusses the impact of key hyperparameters—the reward learning rate $\alpha_{r}$ and λ—on adaptation from both algorithmic and neuroscientific perspectives. We conclude by outlining the limitations of our study and our directions for future research seeking to understand learning algorithms and optimize them further within AI and neuroscience contexts.

## 2. Related Works

### 2.1. Challenges Posed by Noise in RL

Noise and uncertainty are formidable obstacles in RL tasks that directly impact the performance, robustness, and generalizability of the associated learning algorithms [28,29,30,31], while RL methods also inherently struggle with issues such as sample efficiency, scalability, generalizability, and robustness due to their structure. Therefore, a critical challenge in deploying RL in real-life scenarios is its sensitivity to noisy observations, which are a ubiquitous issue given that real-world sensing technologies are intrinsically imperfect [28,32,33].

Value-based RL methods such as Q-learning are especially vulnerable to these noisy observations [34,35,36]. Noise in the observed data can significantly compromise the stability and accuracy of the estimated Q-values, particularly during the early learning stages, during which phase an agent is still developing its policy decisions based on these preliminary estimates. The small sample sizes typical of early learning exacerbate this issue, as they can bias the estimates and lead to suboptimal decision-making. Inaccuracy hinders the convergence of the learning process and occasionally requires a reset to the potentially more accurate initial state [35].

A recent empirical study comparing the efficiency of Q-learning, Q(λ)-learning, SF learning, and PF learning under noisy conditions highlighted significant variability in their performance across one- and two-dimensional gridworlds [27]. Its findings indicated that the efficiency of these algorithms in noisy environments is influenced non-linearly by the λ parameter, suggesting a complex relationship between the degree of noise and the learning efficiency. These observations underscore the need for further research into the resilience of RL algorithms in the face of environmental noise and uncertainty, particularly in terms of their ability to maintain learning efficiency and stability.

### 2.2. Enhancing RL with Transfer Learning

Transfer learning proves particularly effective in addressing the sample inefficiency issues prevalent in traditional RL algorithms [2]. Through utilizing prior knowledge, it enables agents to learn and adapt to new tasks more swiftly and with reduced dependency on extensive datasets and computational resources—a boon in scenarios where acquiring data is costly or impractical [5].

The exploration of transfer learning continues to evolve, especially with the integration of deep learning methods. Recent surveys have discussed the vast progress in deep RL and the pivotal role of transfer learning in solving complex sequential decision-making problems by leveraging external expertise to augment the RL process [37]. For example, Barreto et al. [14] presented a novel framework utilizing “successor features” to enable transfer across tasks with differing reward functions but identical environmental dynamics, highlighting the strategic separation of dynamics from rewards. Additionally, Varma et al. [38] applied pre-trained deep networks like ResNet50 to enhance the efficiency of RL tasks and showcased significant improvements in performance in multi-task scenarios.

### 2.3. Hyperparameter Optimization in RL

Hyperparameter optimization is indispensable in RL, as it directly influences the performance, efficiency, and robustness of learning algorithms [39]. Hyperparameters are the adjustable parameters within them that control various aspects of the learning process, such as learning rates, exploration–exploitation trade-offs, and regularization, with fine-tuning, significantly impact an algorithm’s ability to learn effectively and generalize to new environments or tasks [2].

Given the key role of hyperparameter optimization, researchers have developed a variety of approaches and techniques for selecting the most suitable hyperparameter configurations in RL, seeking to minimize the need for manual tuning and boost the performance of agents in transfer learning and adaptation tasks. Grid search, random search, and Bayesian optimization are among the most popular optimization methods for these purposes.

Grid search and random search are commonly applied to exploring the hyperparameter space [40]. Grid search is a simple but exhaustive approach to hyperparameter tuning that involves evaluating all possible combinations of hyperparameter values within a predefined range [40]. Although it is computationally expensive, grid search ensures that the best combination of hyperparameters is identified. However, its performance may be limited by the granularity of the search space and the availability of computational resources. Random search, on the other hand, randomly samples hyperparameter values from a defined distribution, reducing the computational burden associated with grid search while still maintaining a high probability of identifying suitable combinations [40]. When only a small subset of hyperparameters significantly affect the performance, this method is particularly useful for quickly exploring the most impactful ones.

More advanced techniques, such as Bayesian optimization and evolutionary algorithms, have emerged as promising alternatives for hyperparameter optimization in RL [41]. In leveraging a probabilistic model to guide the search process, Bayesian optimization facilitates more efficient exploration of the hyperparameter space. Meanwhile, evolutionary algorithms employ mechanisms inspired by natural selection and genetic variation to iteratively optimize the hyperparameters. These methods have demonstrated success in identifying the optimal hyperparameter configurations and improving the performance of RL algorithms across various domains and tasks.

## 3. Materials and Methods

### 3.1. Markov Decision Processes

In this study, we operate under the assumption that RL agents engage with their environment through MDPs. The MDPs employed in this research are represented by the tuple M:=(S,A,R,γ), which encompasses the following components. Set S denotes the states, encapsulating information pertaining to the environment, set A signifies the range of actions that an agent is capable of, function R(s) assigns the immediate reward obtained in specific state *s*, and the discount, factor γ∈[0,1), is responsible for controlling the value attributed to future rewards.

The primary objective of the RL agent is to optimize the policy function so that it maximizes the cumulative discounted reward or return, denoted as $G_{t}=\sum_{i=t}^{\infty }\gamma^{i-t}r_{i+1}$, where the immediate reward $r_{t}$ is a function of the state $s_{t}$. To tackle this challenge, dynamic programming techniques are employed to define and calculate a value function $V^{\pi }\left(s\right):=E^{\pi }\left[G_{t}|s_{t}=s\right]$, reliant on the policy chosen, π. This value function is approximated by a function $v^{w}\left(s\right)\approx v^{\pi }\left(s\right)$, which is parameterized by a weight vector $w\in R^{d}$. This weight vector is updated using TD learning as follows: $w_{t+1}=w_{t}+\alpha \left[r_{t+1}+\gamma v_{w}\left(s_{t+1}\right)-v_{w}\left(s_{t}\right)\right]\nabla_{w}v_{w}\left(s_{t}\right)$.

TD(0) refers to an algorithm that employs the one-step TD update rule mentioned above. In contrast, TD(λ) utilizes an eligibility trace to integrate past experiences into the update process. The update mechanism for TD(λ) is expressed as $w_{t+1}=w_{t}+\alpha \delta_{t}e_{t}$, where $\delta_{t}=r_{t+1}+\gamma v_{w}\left(s_{t+1}\right)-v_{w}\left(s_{t}\right)$ represents the TD error, and $e_{t}=\gamma \lambda e_{t-1}+\nabla_{w}v_{w}\left(s_{t}\right)$ denotes the eligibility trace, with λ serving as the decay parameter for the trace.

### 3.2. Predecessor Feature Learning

In this section, a brief introduction to the implementation of PFs in RL algorithms is provided. Given that this topic has been thoroughly discussed in previous papers [19,27], here, we present a concise overview of their main points and their relevance to the current study.

The core idea in SF and PF learning is that the value function $V^{\pi }$, which depends on a policy function π, can be decomposed into the expected visiting occupancy $I(S=s^{\prime })$ and the reward of the successor state $R(s^{\prime })$ as follows:

(1) $$\begin{array}{cc} V^{\pi }\left(s\right) & =E^{\pi }\left[\sum_{i=t}^{\infty }\gamma^{i-t}R\left(s_{i+1}\right)|s_{t}=s\right]\\ \; & =\sum_{s^{\prime }}E^{\pi }\left[\sum_{i=t}^{\infty }\gamma^{i-t}I\left(s_{i}=s^{\prime }\right)R\left(s^{\prime }\right)|s_{t}=s\right]\\ \; & =\sum_{s^{\prime }}M\left(s,s^{\prime }\right)R\left(s^{\prime }\right) \end{array}$$

where the matrix M, which is referred to as the SR, symbolizes the discounted expectation of transitioning from a given state *s* to its successor $s^{\prime }$.

To estimate M(s,:) and $R(s^{\prime })$, we can employ weight matrices and vectors to factorize them as $M\left(s,:\right)=W\phi \left(s\right):=\psi^{W}\left(s\right)$ and $R\left(s^{\prime }\right)=\phi \left(s^{\prime }\right)\cdot w^{r}$. The state feature vector ϕ(s) can be depicted as a one-hot vector within the tabular environment $R^{\left|S\right|}$.

Through employing the TD(λ) learning rule, the matrix W can be updated as follows:

(2) $$\begin{array}{cc} W_{t+1} & =W_{t}+\alpha_{W}\left[\phi \left(s_{t}\right)+\gamma \psi^{W}\left(s_{t+1}\right)-\psi^{W}\left(s_{t}\right)\right]⊗e_{t}\\ e_{t} & =\gamma \lambda e_{t-1}+\phi \left(s_{t}\right) \end{array}$$

where $e_{t}$ and $\alpha_{W}$ represent the eligibility trace of the TD(λ) learning rule and the feature weight learning rate, respectively. Instances with λ=0 correspond to SFs, while those with 0<λ<1 pertain to PFs.

To update $w^{r}$ to learn the factorized reward vector R(s), the TD learning rule is employed as follows:

(3) $$w_{t+1}^{r}=w_{t}^{r}+\alpha_{r}\left(R_{t}-\phi \left(s\right)\cdot w_{t}^{r}\right)\phi \left(s\right)$$

where $\alpha_{r}$ represents the learning rate for the reward vector. 

### 3.3. Experimental Design for Transfer Learning

**Figure 1:**
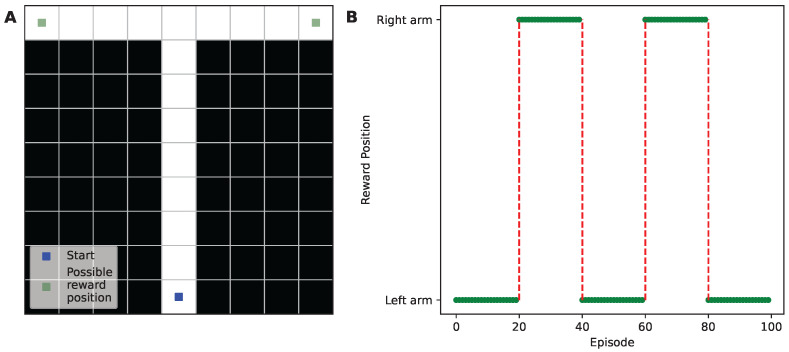
Schematic representation of the T-maze environment and experimental setup. (**A**) The T-maze structure, illustrating the layout and the possible reward locations. (**B**) A plot depicting how the pattern of reward location changes every 20 episodes over a total of 100 episodes, highlighting the dynamics of the environment experienced by the agent. The green dots represent the specific reward position in each episode, while the red dashed lines illustrate the shift of reward location, alternating from left to right or vice versa.

To evaluate the efficacy of the PF learning algorithm for transfer learning, we devised a T-maze environment with noisy observation vectors. The T-maze was implemented within a 9 × 9 grid world, with the agent beginning at the bottom center (Figure 1A). An episode ends when the agent obtains a reward of 1, situated at the end of either the left or right arm. A minimum of 12 steps are required for the agent to acquire a reward, and if it reaches 500 steps, the episode ends with a reward of 0. To examine the effectiveness of transfer learning in this T-maze environment, we alternated the reward’s position to the opposing arm every 20 episodes (Figure 1B). The agent’s action space comprised four possible actions: moving up, down, left, or right.

### 3.4. Gaussian Noise in State Observations

**Figure 2:**
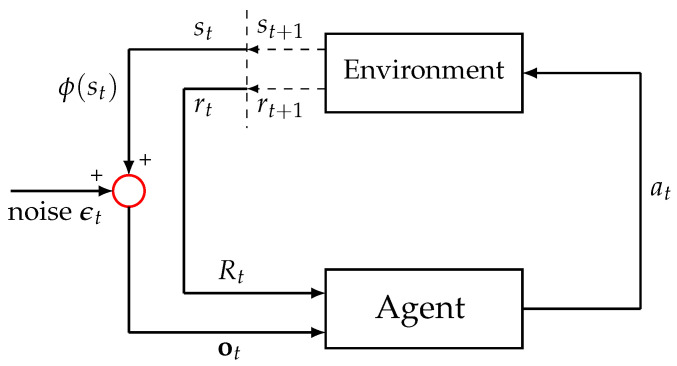
A visual depiction of the interaction between the environment and the agent in reinforcement learning with noisy observations. The environment first updates its state to $s_{t+1}$ and determines the subsequent reward ($r_{t+1}$) following the agent’s action $a_{t}$ at time step *t*. The updated state and reward are then relayed to the agent, alongside an observation $o_{t}$ influenced by the current state. A noise term $\epsilon_{t}$ is applied to the state representation $\phi (s_{t})$, modifying the observation before it is provided to the agent. The red circle highlights the addition of a Gaussian noise vector to the state vector, forming the observation vector. The agent receives an observation $o_{t}$ and a reward $R_{t}$ at each time step *t*, based on which it decides on an action $a_{t}$.

A one-hot state feature vector $\phi (s_{t})$ representing the agent’s current state was provided (Figure 2). Given the common occurrence of sensor noise in real-world contexts, to increase the complexity and reality of the T-maze environment, we introduced noise into $\phi (s_{t})$. As previously described [27], we incorporated noise solely into the state observations while keeping the state transition dynamics in the environment unchanged. A Gaussian noise term was added to the observation vector $o_{t}$ received by the agent in each state as follows:

(4) $$o_{t}=\phi \left(s_{t}\right)+\epsilon_{t},\;\epsilon_{t}∼N\left(0,\sigma^{2}I\right)$$

where $\epsilon_{t}$ denotes a Gaussian noise vector with a mean of zero and the covariance matrix $\sigma^{2}I$, and I represents the identity matrix. Our prior research indicates that excessive levels of noise can obscure discernible differences in performance between agents [27]. Consequently, in this study, we set the noise parameter σ to 0.05, a level previously established as low, to ensure that the comparative analysis was clear.

The feature vectors ϕ(s) utilized in Equations (2) and (3) for the learning PFs were transformed into observation vectors $o_{t}$, allowing the PF algorithm to be described in Algorithm 1.
**Algorithm 1** Predecessor feature learning with noisy observations1:**procedure** PF($episodes,W^{pf},w^{r},\lambda ,\alpha_{W},\alpha_{r}$)2:     initialize $W^{pf},w^{r}$3:     **for** episode in 1…n **do**4:        $s_{t}\leftarrow$ initial state of episode5:        e←0 (eligibility trace reset)6:        **for** pair ($o_{t}$,$o_{t+1}$) and reward *r* in episode **do**7:            $e\leftarrow e+o_{t}$8:            $\delta^{pf}\leftarrow o_{t}+\gamma W^{pf}\cdot o_{t+1}-W^{pf}\cdot o_{t}$9:            $\delta^{r}\leftarrow r-o_{t+1}\cdot w^{r}$10:          $W^{pf}\leftarrow W^{pf}+\alpha_{W}\delta^{pf}⊗e$11:          $w^{r}\leftarrow w^{r}+\alpha_{r}\delta^{r}o_{t+1}$12:          e←γλe13:    **return** $W^{pf},w^{r}$

In line with our previous finding that random initialization improves the learning efficiency, $W^{pf}$ was initialized using random variables [42].

### 3.5. Hyperparameters of the Algorithm

In order to assess the influence of the hyperparameters on the agent’s transfer learning capabilities in a noisy T-maze environment, specifically λ and $\alpha_{r}$, a series of 100 trials were conducted, with each encompassing 100 episodes. We selected the hyperparameters λ and $\alpha_{r}$ due to their crucial roles in shaping the adaptive behavior of RL agents, particularly in noisy environments. The eligibility trace decay rate λ controls how past states and actions influence the current learning through credit assignment over time. Preliminary experiments and our previous findings [27] demonstrated that varying λ significantly affects the performance of the agent in noisy conditions, enhancing its adaptability by appropriately balancing the influence of past experiences. The reward learning rate $\alpha_{r}$ dictates the pace at which the agent updates its reward expectations, balancing learning speed with stability. Effectively tuning $\alpha_{r}$ is vital in noisy environments: Lower values stabilize learning by reducing the sensitivity to noise, while higher values enable quicker adaptation to changing reward patterns.

We examined five distinct hyperparameter configurations, with λ∈{0,0.2,0.4,0.6, and 0.8} and $\alpha_{r}\in \{0.1,0.3,0.5,0.7,$ and 0.9}. The transfer learning efficacy of 25 unique agents was analyzed by employing unique combinations of these two hyperparameters. Notably, λ=0 represents the SF algorithm, with which the learning is not influenced by an eligibility trace and the immediate state–action–reward relationships are effectively isolated. This setting provides a baseline against which the impact of eligibility traces can be assessed [14]. Additionally, these parameter ranges facilitate the robust exploration of how varying the level of credit assignment (λ) and the learning rate ($\alpha_{r}$) affects the agent’s adaptability, particularly under noisy conditions. Studies have shown that by adjusting these hyperparameters, a trade-off between learning speed and stability is achieved, and complex dynamic environments can be managed, in which both the state transitions and reward structures can vary significantly [15]. This structured approach using grid search at the normal scale rather than a logarithmic scale ensures precise tuning, enhancing the overall model performance by allowing the effects of incremental parameter adjustments to be directly and systemically evaluated.

In our experiments, the ϵ-greedy policy was implemented as the agent’s policy function. This policy alternates between exploring random actions with a probability of ϵ and exploiting known actions with the highest Q-value estimate with a probability of 1−ϵ. To achieve a balance between exploration and exploitation, the probability of ϵ decayed over episodes according to $\epsilon_{k+1}=0.9\cdot 0.8^{t}+0.1$, where *k* was the episode index, while the feature weight learning rate $\alpha_{W}$ and the discount factor γ were set to 0.1 and 0.95, respectively.

### 3.6. Evaluation Metrics for Transfer Learning

In this study, we employed four evaluation metrics to assess the performance of agents trained with different hyperparameters in transfer learning. These metrics were as follows:

1. Cumulative reward: The total reward accumulated by the agent over the course of the episodes, which serves as an indicator of the agent’s overall performance in the task.
2. Step length to the end of the episode: The number of steps it took the agent to reach the end of an episode, reflecting its ability to efficiently navigate the environment.
3. Adaptation rate: An indicator of the number of episodes an agent took to adapt to the reward location being switched. The adaptation rate was calculated by assessing the episodes in which the agent successfully reached the reward location five times consecutively, which were then considered in determining the adaptation rate. For example, if an agent reached this performance level by the 10th episode after the reward location shifted, its adaptation rate would equate to 10, meaning the optimal adaptation rate was defined as 5. A lower adaptation rate implies swifter adaptation of the agent to the new reward location, whereas a higher adaptation rate denotes a more gradual process of adjustment.
4. Adaptation step length: This measure evaluates the number of steps required by an agent to reach its defined adaptation rate. For instance, with the adaptation rate set at 10, this metric calculates the steps necessary to successfully navigate 10 episodes following a change in the reward’s location. Utilizing a calculation approach akin to that for the adaptation rate but focused on steps rather than episodes, this metric offers an alternative view of an agent’s ability to adjust to environmental changes.

In this study, we examined the relationships between each evaluation metric and the hyperparameters, particularly λ and $\alpha_{r}$, using the Spearman’s correlation analysis tool from the SciPy library (version 1.7.3) [43] and the linear regression model from the statsmodels library (version 0.13.5) [44].

## 4. Experimental Results

In our investigation, we evaluated transfer learning for agents with different hyperparameters in a noisy T-maze space (a 9 × 9 grid) in which the reward locations changed every 20 episodes. We tested 25 agents across 100 runs of 100 episodes each, with their λ values varying from 0 to 0.8 and their reward learning rate $\alpha_{r}$ values varying from 0.1 to 0.9 (see Section 3 for details). Those for which λ=0 were SFs, and the rest were PFs.

### 4.1. Analyzing the Cumulative Reward and Step Length

#### 4.1.1. Cumulative Reward

**Figure 3:**
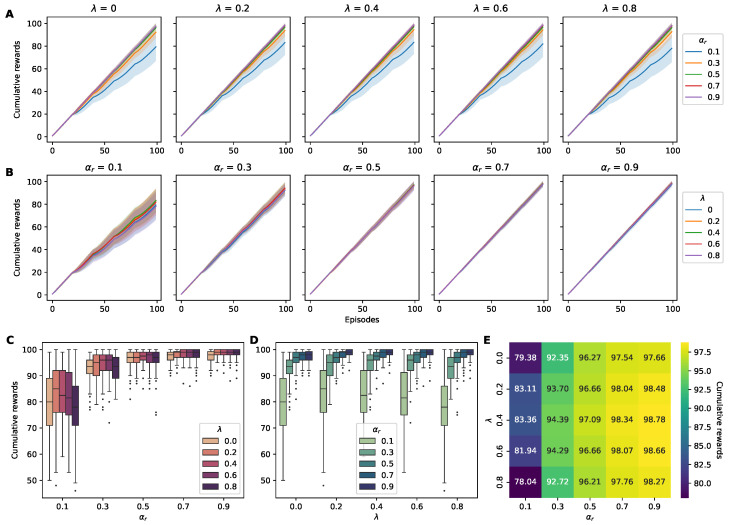
Cumulative reward metrics for RL agents across hyperparameter variations. (**A**) Five panels displaying the cumulative rewards obtained by agents with different λ values over 100 episodes, with each panel containing color-coded plots representing changes in $\alpha_{r}$ (0.1: blue; 0.3: orange; 0.5: green; 0.7: red; and 0.9: purple). The shaded area around the lines indicates the standard deviation across runs. (**B**) Five panels displaying cumulative rewards for varying learning rates $\alpha_{r}$, with each panel containing color-coded plots representing the changes in λ (0: blue; 0.2: orange; 0.4: green; 0.6: red; and 0.8: purple). (**C**,**D**) Box plots of the cumulative rewards for all combinations of λ and $\alpha_{r}$, highlighting the distribution and central tendency of the rewards collected by agents. (**E**) A heatmap illustrating the cumulative reward for each combination of $\alpha_{r}$ and λ, highlighting the optimal hyperparameter settings for maximizing reward.

Upon examining the data on the cumulative reward for each agent, we observed a correlation between the hyperparameters and the agents’ performance: Agents with a higher reward learning rate $\alpha_{r}$ tended to obtain a higher cumulative reward (Figure 3). In general, the SF agents (λ=0) obtained lower cumulative rewards compared to the PF agents. However, there was not a consistent positive correlation for λ akin to that for the reward learning rate $\alpha_{r}$. The optimal hyperparameter combination was α=0.9 and λ=0.4, with an average reward of 98.78 (Figure 3E).

Analyses based on Spearman’s correlation and linear regression were conducted to investigate the relationship between the hyperparameters and the cumulative reward further. Spearman’s analysis showed a significant positive correlation between the reward learning rate $\alpha_{r}$ and the cumulative reward, while the correlation coefficient between λ and the cumulative reward did not indicate significance (Table 1). The same trends emerged in applying linear regression to the results (Table 1).

#### 4.1.2. Step Length

**Figure 4:**
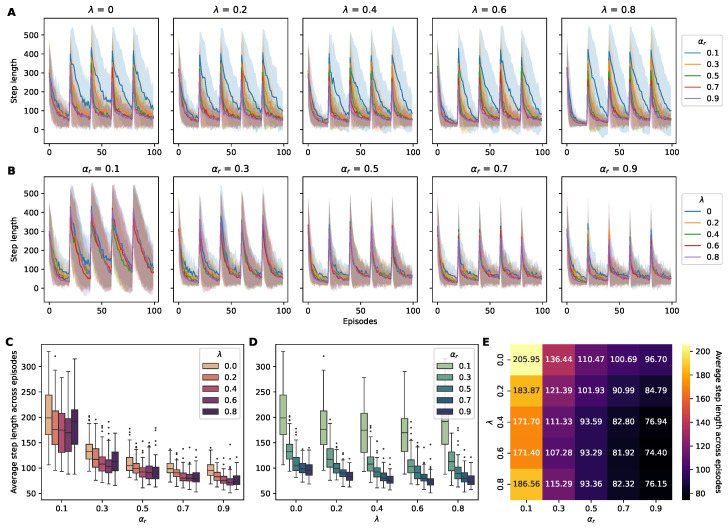
Step lengths of RL agents across hyperparameter variations. (**A**) Five panels displaying the step lengths with different λ values over 100 episodes, with each panel containing color-coded plots representing changes in $\alpha_{r}$ (0.1: blue; 0.3: orange; 0.5: green; 0.7: red; and 0.9: purple). The shaded area around the lines indicates the standard deviation across runs. (**B**) Five panels displaying the step lengths for a varying learning rate $\alpha_{r}$, with each panel containing color-coded plots representing the changes in λ (0: blue; 0.2: orange; 0.4: green; 0.6: red; and 0.8: purple). Note that every 20 episodes, the step length increases due to the repositioning of the reward. (**C**,**D**) Box plots of the step lengths for all combinations of λ and $\alpha_{r}$, highlighting the distribution and central tendency of the step lengths of the agents. (**E**) A heatmap illustrating the average step length for each combination of $\alpha_{r}$ and λ, highlighting the optimal hyperparameter settings that result in the lowest step lengths.

To assess the agents’ efficiency in navigating the dynamic T-maze environment, the metric of the step length to the end of the episode was employed. Lower step lengths indicate more efficient agents capable of rapidly reaching the altered reward location. Our simulations demonstrated an average step length exceeding 400 when the reward location changed, followed by a rapid decrease (Figure 4).

In assessing the efficiency of learning and exploration, the step lengths of the 25 agents were averaged over 100 episodes. Contrary to the trend in the cumulative reward observations, the agents with hyperparameters of $\alpha_{r}=0.9$ and λ=0.6 demonstrated the most efficient performance, achieving the shortest average step lengths, as depicted in Figure 4E. A consistent decrease in the average step length was observed with an increasing reward learning rate $\alpha_{r}$, with a less pronounced trend for the λ values. Spearman’s correlation corroborated this trend, revealing a significant negative correlation between the reward learning rate $\alpha_{r}$ and step length and a subtle negative correlation of step length with λ (Table 1). The linear regression analysis also indicated a notable inverse relationship between the reward learning rate ($\alpha_{r}$) and step length, suggesting that higher learning rates correlated with reduced step lengths. This trend was consistent with the cumulative reward outcomes. However, the analysis for λ diverged, showing no significant correlation with cumulative reward. (Table 2).

### 4.2. Analysis of the Adaptation Metrics

In this study, we evaluated the agents’ adaptability to changes in the reward location within the T-maze environment using two adaptation metrics (see Section 3.6 for details).

#### 4.2.1. Adaptation Rate

**Figure 5:**
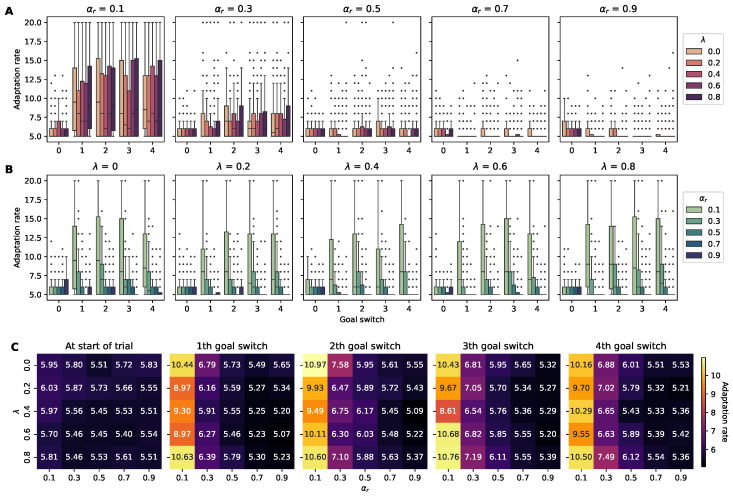
Rates of adaptation of RL agents to changes in the reward’s position across different hyperparameter settings. (**A**) Five panels separated by $\alpha_{r}$ with color-coded box plots of the adaptation rates for agents with varying λ values across four consecutive goal switches. (**B**) Panels divided according to λ with color-coded box plots of the adaptation rates for agents with varying $\alpha_{r}$ levels. Each box plot represents the interquartile range of the adaptation rates observed over 100 trials, with outliers shown as individual points. (**C**) A set of heatmaps displaying the adaptation rate at the start of trials and after each switch in the reward location, color-coded for visual comparison across the combinations of the hyperparameters $\alpha_{r}$ and λ.

The adaptation rate metric quantifies the number of episodes required for an agent to reach the reward location five consecutive times after it changes, where a lower adaptation rate indicates that agents are more adaptable to alterations in the reward location. During a trial consisting of 100 episodes, the reward relocates a total of four times. Similar to the findings when analyzing the cumulative rewards, higher $\alpha_{r}$ levels were correlated with a reduced adaptation rate, whereas no significant association was observed between λ and the adaptation rate (Figure 5). The optimal hyperparameter configurations required to achieve the lowest adaptation rate varied with each change in the position of the reward. While a combination of $\alpha_{r}=0.9$ and λ=0.6 yielded the best adaptation to the first shift in the reward’s position, the effectiveness of this initial configuration diminished for subsequent changes in its position. Specifically, after the fourth change, the configuration of $\alpha_{r}=0.9$ and λ=0.2 emerged as the most effective, indicating a shift in the optimal hyperparameter settings for an agent to adapt to the reward being in a new position.

The results from the Spearman’s correlation test indicated a statistically significant negative correlation between the learning rate $\alpha_{r}$ and the adaptation rate, suggesting that increases in $\alpha_{r}$ may lead to decreases in the adaptation rate (Table 3). λ was also negatively correlated with the adaptation rate, but this association was considerably weaker, as evidenced by its correlation coefficient. The linear regression analysis identified a significant negative correlation between the learning rate $\alpha_{r}$ and the adaptation rate, indicating that higher $\alpha_{r}$ values are associated with decreased efficiency in adaptation (Table 4). In contrast, the analysis found no significant correlation between λ and the adaptation rate, suggesting that λ has a minimal impact on the adaptation performance.

#### 4.2.2. Adaptation Step Length

The metric of the adaptation step length measures the total step lengths required by an agent within episodes to achieve the adaptation rate threshold. Our initial findings indicated that agents with a learning rate ($\alpha_{r}$) of 0.9 and a trace decay (λ) of 0.6 required the fewest steps to adjust after the first change in the position of the reward. However, as the environment evolved, the configuration with $\alpha_{r}=0.9$ and λ=0.2 emerged as the most adept at minimizing adaptation steps following the fourth shift in the position of the reward.

The Spearman’s correlation analysis indicated that there was a statistically significant negative correlation between the learning rate $\alpha_{r}$ and the adaptation step length, suggesting that as $\alpha_{r}$ increased, the adaptation step length decreased. However, λ was much more weakly correlated with the adaptation step length, suggesting that λ has a less direct effect. The subsequent linear regression analysis confirmed this pattern, showing a significant negative association between $\alpha_{r}$ and the adaptation rate but no such association for λ.

Taken together, these results suggest that while the learning rate $\alpha_{r}$ is critical to the rate of adaptation, the impact of λ is less clear-cut, and further investigation is needed to understand its role in the adaptation process.

## 5. Conclusions and Future Work

The present study systematically investigated the effects of different hyperparameter values on the performance, efficiency, and adaptation of agents in the context of transfer learning in a T-maze environment. Our results revealed that higher $\alpha_{r}$ levels were consistently associated with improved cumulative rewards and adaptation metrics, while varying the λ values had a less consistent influence across different metrics.

In this section, we aim to (1) discuss the impact of the hyperparameters on agent adaptability and performance, delving into the role of $\alpha_{r}$ and λ in modulating the trade-off between learning efficiency and effective forgetting; (2) delve into the neuroscientific implications of our findings, drawing parallels between the mechanisms that underpin the performance of agents in RL and biological learning processes; (3) acknowledge the limitations of the current study and propose avenues for future research; and (4) summarize the key takeaways from this study.

### 5.1. Hyperparameters

As may be evident, our findings have demonstrated that a higher learning rate $\alpha_{r}$ contributes to enhanced efficiency in learning transitions because it centers around learning the locations of the reward. On the other hand, the eligibility trace parameter, λ, in retrospectively updating the future occupancy of the state, has a more nuanced impact in a simple T-maze environment. This is because even when the reward position switches to the other arm, the same state transition matrix applies until the agent reaches the bifurcation from which the arms lead. This observation highlights the importance of considering the specific characteristics of the learning environment when interpreting the influence of hyperparameters on agent performance. In more complex environments, the interplay between $\alpha_{r}$ and λ might differ, and therefore, the best combination of hyperparameters for maximizing the learning efficiency and transfer capabilities may also change [2].

**Figure 6:**
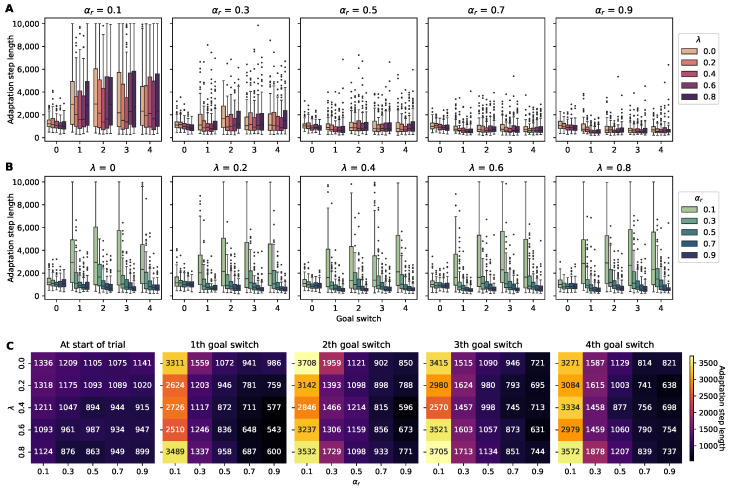
Adaptation step lengths of RL agents for changes in the position of the reward across different hyperparameter settings. (**A**) Five panels separated by $\alpha_{r}$ with color-coded box plots of the adaptation step lengths for agents with varying λ values across four consecutive switches in the reward. (**B**) Panels divided according to λ with color-coded box plots of the adaptation step lengths for agents with varying $\alpha_{r}$ levels. Each box plot represents the interquartile range of the adaptation step lengths observed over 100 trials, with outliers shown as individual points. (**C**) A set of heatmaps displaying the adaptation step lengths at the start of trials and after each switch in the location of the reward, color-coded for visual comparison across the combinations of the hyperparameters $\alpha_{r}$ and λ.

The first panel in Figure 6C indicates that higher λ values initially boost the learning efficiency before the reward’s location changes. This could be interpreted as λ allowing the state values to be forgotten more slowly when its location shifts, which may be disadvantageous in terms of adaptability in dynamic settings. Given that effective forgetting is crucial for flexible learning [45], it is plausible that elevated λ values might hinder adaptability by impeding the process of forgetting, and this hypothesis is supported by the observation that after multiple relocations of the reward, the optimal performance involves lower λ values.

Our findings position the role of λ in harmonizing between adaptability and the retention of previously acquired information as a prime concern for the development of more efficient learning algorithms in the future. In this respect, research investigating the performance of PF and SF learning algorithms in more diverse and challenging environments could advance our understanding of the ability of hyperparameter optimization to inform effective learning and adaptability.

Indeed, this study contributes significantly to our understanding in the context of T-maze environments by systematically examining the effects of varying the hyperparameter values on agent performance and adaptation. Our results corroborate the emphasis in previous studies of the importance of learning rates to RL tasks [2], where higher rates facilitate rapid updates to knowledge and adaptability to environmental changes. However, the impact of λ on agent performance appears to be less consistent, suggesting that eligibility traces affect the learning outcomes differently depending on the complexity of the task and the structure of the environment at hand.

### 5.2. Neuroscientific Implications

This investigation sought to elucidate the efficacy of transfer learning within PF and SF learning algorithms, particularly in terms of their alignment with the neural processes that have been uncovered within the mammalian brain, and contribute to the development of biologically inspired AI algorithms [13,46,47].

Previous research has demonstrated that mammalian brains employ RL mechanisms for decision-making, learning, and adaptation [48,49,50,51]. In particular, the dopamine system plays a crucial role in indicating errors in reward predictions and modulating synaptic plasticity [52,53]. Similarities between the TD learning algorithm that underpins RL in artificial agents and the functioning of the dopaminergic system in the brain have also been identified [54,55,56,57,58]. In this study, the TD learning algorithm involved a balance between PFs and SFs, and the relationship determined between the hyperparameters ($\alpha_{r}$ and λ) and agent performance may offer insights into the optimal balance of neural processes that facilitate efficient learning and adaptation in biological systems as well.

One study recently published by Bono et al. [59] provided a compelling explanation of how the TD(λ) algorithm is implemented biologically in the hippocampus through synaptic spike-timing-dependent plasticity (STDP). Their insights shed light on our study’s findings specifically as they further elucidate the role of the λ hyperparameter in learning and adaptation. Bono et al. [59] discussed the relationship between λ and various biological parameters, including state dwell times, neuronal firing rates, and neuromodulation. Interestingly, in alignment with psychological studies but in contradiction to conventional RL theory, their study suggests that the discount factor decreases hyperbolically over time. When considered in conjunction with our findings, a potential relationship emerges between the λ hyperparameter’s role in learning and adaptation and the time constant in STDP. In our study, it seemed that the λ hyperparameter may play a similar role in balancing efficiently retaining and forgetting information, with higher values promoting faster retrospective updates but potentially hindering effective forgetting. The neurobiological underpinnings of STDP could therefore be instrumental in balancing between efficient learning and the effective erasure of obsolete information.

As regards the biological implementation of the TD(λ) rule, synaptic eligibility traces are well-known phenomena [60]. Higher λ values facilitate more extensive retrospective updates to the state, which could be interpreted as extensions of the eligibility traces over time. Additionally, if we assume that these eligible synapses are updated in response to the arrival of reward signals such as dopamine [61], this aligns with the findings for the $\alpha_{r}$ parameter in our study. Consequently, we postulate that plasticity updates, contingent upon eligibility tracing and the reward, are tied to the balance between λ and $\alpha_{r}$. Future inquiries could delve deeper into these interconnections to foster the development of more biologically congruent AI algorithms and augment our comprehension of the brain’s learning mechanisms.

In addition to the mechanisms of the dopaminergic system, our findings may also inform our understanding of the role of the hippocampus in spatial learning and memory. The hippocampus is a brain structure that has been implicated in forming and retrieving spatial memories, as well as in encoding contextual information [6]. The PFs and SFs used in our study may be analogous to the neural representations of spatial context and future states that are formed in the hippocampus [12,13,59,62,63]. Thus, the relationships observed between the hyperparameters and agent performance in our T-maze task could have implications for the neural processes that underlie the formation of and updates to spatial memories and context-dependent decision-making.

### 5.3. Limitations and Future Research Directions

Although this study uncovered valuable insights into the performance of PF and SF learning algorithms in a T-maze environment and the optimal hyperparameters for this context, it also had several limitations. Firstly, the T-maze environment used is relatively simplistic and may not reflect the complexity involved in real-world tasks, which limits the generalizability of our findings. Moreover, we concentrated on a noise level of 0.05, which may not represent the diverse levels of uncertainty inherent in different domains, thereby complicating the application of our results to other environments and noise levels [27]. Additionally, given that our study explored a limited range of combinations of $\alpha_{r}$ and λ, other unexplored combinations might yield different insights or even a superior agent performance. Lastly, not all aspects of agent adaptability and learning were covered in that we focused on four evaluation metrics. Including alternative or additional metrics, particularly those related to transfer learning and adaptation, may unveil further distinctions within the learning process.

We focused on evaluating the performance of SFs and PFs within a noisy T-maze environment in this study. In our previous work, SFs and PFs were analyzed and compared against traditional RL methods, such as Q-learning and Q(λ) learning, with SFs and PFs outperforming these methods in noisy environments [27]. These findings highlight the advantages of SFs and PFs in scenarios where traditional algorithms struggle due to the added complexity of noise. While advanced deep learning methods, including transformers and GANs, have shown significant promise in handling noise [25,26], they are not directly applicable to our experimental setup due to the discrete, grid-based nature of the T-maze. They have typically been designed for high-dimensional, continuous-state spaces, which offer more variability and complexity than the structured, low-dimensional T-maze environment. Consequently, methods such as SFs and PFs were more suitable for our task. Despite the constraints of our current comparison, these limitations provide avenues for future research. Transformers and GANs could be integrated and evaluated by expanding our approach to more complex RL environments, uncovering the potential of these advanced deep learning algorithms to enhance adaptability and performance in more sophisticated settings and broadening the applicability of our findings.

This study’s findings serve as a springboard for future research seeking to ascertain the robustness of PF and SF learning algorithms within a wider variety of environments, from complex grid worlds to autonomous vehicles. Developing metrics using concepts such as path optimality or the development of latent cognitive maps could deepen our understanding of agent performance. Furthermore, extending the range of hyperparameters and utilizing optimization techniques such as grid search, random search, or Bayesian optimization could uncover combinations that promote superior learning and adaptability [40,41,64].

High-fidelity neurobiological models could be employed to examine their influence on agent performance, whether applying spiking neural network models or incorporating plasticity rules, such as STDP, into the modeling process [13,59,62,63]. This approach could lead to a more profound comprehension of neurobiological learning processes, laying the groundwork for in-depth investigations into the role of neuromodulators like dopamine and serotonin in enhancing adaptability and flexibility [65,66,67].

### 5.4. Conclusions

This study elucidated the role of hyperparameters in augmenting transfer learning and adaptation in a T-maze environment. By delving into the correlation between hyperparameters and evaluation metrics, we have enhanced our comprehension of the determinants that facilitate effective learning and adaptation in AI and machine learning.

Specifically, we observed a positive correlation between elevated $\alpha_{r}$ values and higher cumulative reward and adaptation metrics, while the effect of λ appeared to be negligible. Conversely, our findings suggest that as reward repositioning progresses, high λ values hinder the acquisition of new knowledge. These observations may aid in the selection and optimization of the hyperparameters when fine-tuning reinforcement learning algorithms.

In bringing biological insights into transfer learning, memory, and efficient forgetting processes into play, our findings also extend beyond AI. This could pave the way for novel directions within the research on cognitive disorders such as obsessive–compulsive disorder by improving our algorithmic understanding of learning and memory and their connection to cognitive flexibility.

## Figures and Tables

**Table 1:** Spearman’s rank correlation analysis of the relationships between hyperparameters, cumulative rewards, and step length.

Metric	Hyperparameter	Correlation Coefficient ($r_{s}$)	*p*-Value
Cumulative reward	$\alpha_{r}$	0.661	<0.001
	λ	0.034	0.09
Step length	$\alpha_{r}$	−0.750	<0.001
	λ	−0.231	< 0.001

**Table 2:** Linear regression analysis of the relationship between hyperparameters, cumulative rewards, and step length.

Metric	Hyperparameter	Regression Coefficient	95% Conf. Interval
Cumulative reward	(constant)	83.825	83.159, 84.490
	$\alpha_{r}$	19.434	18.483, 20.385
	λ	−0.077	−1.028, 0.874
Step length	(constant)	182.678	179.554, 185.801
	$\alpha_{r}$	−117.405	−121.867, −112.942
	λ	−24.782	−29.244, −20.320

**Table 3:** Spearman’s rank correlation analysis of the relationships between hyperparameters, adaptation rate, and adaptation step length.

Metric	Hyperparameter	Correlation Coefficient ($r_{s}$)	*p*-Value
Adaptation rate	$\alpha_{r}$	−0.363	<0.001
	λ	−0.029	0.001
Adaptation step length	$\alpha_{r}$	−0.428	<0.001
	λ	−0.068	<0.001

**Table 4:** Linear regression analysis of the relationship between hyperparameters, adaptation rate, and adaptation step length.

Metric	Hyperparameter	Regression Coefficient	95% Conf. Interval
Adaptation rate	(constant)	8.643	8.520, 8.765
	$\alpha_{r}$	−4.312	−4.488, −4.137
	λ	−0.059	−0.234, 0.116
Adaptation step length	(constant)	2550.657	2489.834, 2611.481
	$\alpha_{r}$	−2300.500	−2387.391, −2213.609
	λ	−70.776	−157.667, 16.115

## Data Availability

The code used to generate the data for this study is available at https://github.com/NeuroAI-PNU/PF-T-maze. For any additional requests or inquiries, please contact the corresponding author.

## Abbreviations

The following abbreviations are used in this manuscript:

MDPs	Markov Decision Processes;
SFs	Successor Features;
PFs	Predecessor Features;
AI	Artificial Intelligence;
RL	Reinforcement Learning;
SR	Successor Representation;
TD	Temporal Difference;
GANs	Generative Adversarial Networks;
STDP	Spike-Timing-Dependent Plasticity.
